# Protective Effect of Creatine Elevation against Ischaemia Reperfusion Injury Is Retained in the Presence of Co-Morbidities and during Cardioplegia

**DOI:** 10.1371/journal.pone.0146429

**Published:** 2016-01-14

**Authors:** Hannah J. Whittington, Debra J. McAndrew, Rebecca L. Cross, Stefan Neubauer, Craig A. Lygate

**Affiliations:** Division of Cardiovascular Medicine, Radcliffe Department of Medicine. Wellcome Trust Centre for Human Genetics, University of Oxford, Oxford, United Kingdom; Université catholique de Louvain, BELGIUM

## Abstract

**Aims:**

Ischaemic heart disease is most prevalent in the ageing population and often exists with other comorbidities; however the majority of laboratory research uses young, healthy animal models. Several recent workshops and focus meetings have highlighted the importance of using clinically relevant models to help aid translation to realistic patient populations. We have previously shown that mice over-expressing the creatine transporter (CrT-OE) have elevated intracellular creatine levels and are protected against ischaemia-reperfusion injury. Here we test whether elevating intracellular creatine levels retains a cardioprotective effect in the presence of common comorbidities and whether it is additive to protection afforded by hypothermic cardioplegia.

**Methods and Results:**

CrT-OE mice and wild-type controls were subjected to transverse aortic constriction for two weeks to induce compensated left ventricular hypertrophy (LVH). Hearts were retrogradely perfused in Langendorff mode for 15 minutes, followed by 20 minutes ischaemia and 30 minutes reperfusion. CrT-OE hearts exhibited significantly improved functional recovery (Rate pressure product) during reperfusion compared to WT littermates (76% of baseline vs. 59%, respectively, P = 0.02). Aged CrT-OE mouse hearts (78±5 weeks) also had enhanced recovery following 15 minutes ischaemia (104% of baseline vs. 67%, P = 0.0007). The cardioprotective effect of hypothermic high K^+^ cardioplegic arrest, as used during cardiac surgery and donor heart transplant, was further enhanced in prolonged ischaemia (90 minutes) in CrT-OE Langendorff perfused mouse hearts (76% of baseline vs. 55% of baseline as seen in WT hearts, P = 0.02).

**Conclusions:**

These observations in clinically relevant models further support the development of modulators of intracellular creatine content as a translatable strategy for cardiac protection against ischaemia-reperfusion injury.

## Introduction

Decades of research investigating strategies that can reduce the damaging effects of ischaemic heart disease have led to the identification of numerous pre-clinical therapeutic targets. Effective treatment of acute myocardial infarction (AMI) involves the rapid reperfusion of the ischaemic myocardium; however reperfusion can paradoxically lead to further myocardial damage known as ischaemia-reperfusion injury (IRI) [1]. Upon reperfusion, the acutely ischaemic myocardium is subjected to several rapid biochemical and metabolic changes including generation of reactive oxygen species, overload of intracellular Ca^2+^ and rapid restoration of physiological pH which interact to mediate cardiomyocyte death through opening of the mitochondrial permeability transition pore (mPTP) [1].

Energetic deficits are one of many confounding factors that can contribute to IRI. The creatine kinase system acts as an energy buffer for the rapid regeneration of ATP when energy demand surpasses supply [2] (e.g. during ischaemia), and deficiency of this system is detrimental to functional recovery following ischaemia [3,4]. Simply increasing creatine intake does not significantly elevate creatine levels in the adult heart [5], and for this reason our laboratory previously created mice that genetically over-express the specific plasma-membrane creatine transporter (CrT). These mice have elevated myocardial creatine [Cr] and phosphocreatine, which protected against IRI in both in vivo and ex vivo models [6].

Despite the successful application of strategies that initiate cardioprotective signalling in the laboratory [7], both pharmacological and non-pharmacological approaches frequently lose efficacy in the clinical setting [8]. This loss of translation was the focus of a recent European Society of Cardiology (ESC) working group position paper, which highlighted the essential use of more complex experimental models to help bridge the gap between bench and bedside [9]. One of the main issues raised was the use of inappropriate pre-clinical cell/animal models in cardioprotection studies. Pre-clinical laboratory investigations should try to reflect the clinical scenario, in which patients presenting with AMI undergoing revascularisation are of both genders and of older age with many co-morbidities, e.g. hypertension, obesity, and left ventricular hypertrophy (LVH) [10].

Moreover, clinical translation will require creatine elevation prior to ischaemic challenge, e.g. before elective surgery. Any benefit should therefore be additive to that afforded by standard cold hyperkalaemic cardioplegia, which electromechanically arrests the heart in diastole to reduce myocardial metabolic demand. While this strategy prolongs tolerance to ischaemia, there remains room for improvement since some degree of reperfusion injury is still observed in the human heart [11].

To address these concerns we therefore performed isolated perfused heart experiments to determine whether the cardioprotective effect observed in young CrT over-expressing mice (CrT-OE) persists in the presence of ‘comorbidities’. Here we demonstrate that post-ischaemic functional recovery was improved commensurate with myocardial creatine levels in ageing hearts and in hearts with compensated LVH. Furthermore, we show for the first time that elevating intracellular creatine is additive to standard hypothermic cardioplegia in promoting functional recovery.

## Materials and Methods

### Animals

This investigation was approved by the Committee for Animal Care and Ethical Review at the University of Oxford and conforms to the UK Animals (Scientific Procedures) Act, 1986, incorporating Directive 2010/63/EU of the European Parliament. Creatine transporter overexpressing mice (CrT-OE) were bred in house and backcrossed with C57BL/6J mice for greater than 10 generations as previously described [6,12], the Tg46 strain and their wild-type (WT) littermates were used for all experimental procedures. A subset of CrT-OE and WT mice were aged in house to 78 ± 5 weeks. Creatine levels in WT and transgenic mice were confirmed in post-mortem LV tissue using HPLC as previously described [13]. All mice were group housed with chow ad libitum which is naturally creatine-free (Teklad global 16% rodent diet, 2916, Harlan UK), in a controlled environment in specific pathogen-free cages under a 12 h light-dark cycle, at 21–22°C.

### Aortic banding surgery

CrT-OE/WT male (n = 21) and female (n = 18) mice (22 ± 1g, aged 21.9 ± 0.8 weeks) were anaesthetised with isoflurane in 100% O_2_, intubated, and a trans-sternal thoracotomy performed. The transverse aorta was dissected and a 7–0 polypropylene monofilament suture (Prolene, W8725) tied against a modified 27-gauge needle as described by Lygate, 2006[14] to produce transverse aortic constriction (TAC) for a period of 2 weeks. Mice were given peri-operative subcutaneous buprenorphine (0.8mg/kg) for pain relief.

### Echocardiography

Echocardiography was used to confirm the degree of left ventricular (LV) hypertrophy following approximately 10 days of band placement. Mice were lightly anaesthetised using 1–1.5% isoflurane, kept warm on a homeothermic blanket and imaged using a VisualSonics Vevo 2100 with 22–55 MHz transducer. B-mode trans-thoracic short-axis images were obtained at the papillary muscle level to measure diastolic myocardial cross-sectional area (CSA) as a marker of LVH and fractional area change (FAC) as a measure of function. A subset of WT mice were imaged before surgery to confirm 2 weeks of TAC was sufficient to cause significant hypertrophy of the LV.

### Ischaemia/reperfusion recovery ex vivo

All mice were anesthetized with sodium pentobarbital (55 mg/kg I.P.) and heparin (300 IU). Hearts were rapidly excised, cannulated and perfused in Langendorff constant pressure mode at 80mmHg with oxygenated (95% O_2_/5% CO_2_) Krebs-Henseleit buffer at 37°C (mM): NaCl 118, KCl 4.7, MgSO_4_.7H_2_O 1.2, NaHCO_3_ 25, KH_2_PO_4_ 1.2, Glucose 11, CaCl_2_.H_2_O 1.8. LV function was assessed in spontaneously beating hearts using a water-filled intraventricular balloon connected to a pressure transducer (ADinstruments Ltd). The left ventricular end-diastolic pressure (LVEDP) was set to 5.4 ± 0.5 mmHg and heart rate (HR) and left ventricular systolic pressure (LVSP) measurements collected. These parameters were used to calculate left ventricular developed pressure (LVDP), (LVSP—LVEDP = LVDP) and subsequently rate pressure product (RPP) in the heart (HR * LVDP = RPP). Function was continually recorded with parameters averaged at 5 to 10 min intervals throughout the ischaemia-reperfusion protocol. Hearts in the TAC and cardioplegia groups were excluded if they did not attain good baseline ex vivo function: exclusion criteria RPP <15000 mmHg*bpm and/or LVDP <50 mmHg at the end of the stabilisation period [15]. Exclusion criteria was lowered to RPP <10000 mmHg*bpm in aged hearts. In addition, hearts were excluded from analysis if they failed to make any functional recovery. This applied to n = 3 WT hearts that did not recover following cardioplegic arrest; inclusion or exclusion of these hearts did not affect our findings. CrT-OE and WT mice were used in the experimental protocols outlined below and the left ventricle subsequently snap frozen in liquid nitrogen and stored at -80°C for subsequent biochemical analysis.

### TAC

Fourteen days post TAC surgery all hearts that were excised (WT; n = 9 female, n = 12 male. CrT-OE; n = 9 female, n = 9 male) had the aortic band visibly intact. Following cannulation, hearts were stabilised for 15 min, subjected to global ischaemia for 20 min and reperfused for 30 min. Four male WT hearts and one male CrT-OE heart did not meet the minimum baseline function criteria and were therefore excluded from the study.

### Ageing

Hearts from aged WT and CrT-OE mice (78 ± 5 weeks) were stabilised for 15 minutes, subjected to global ischaemia for 15 minutes and reperfused for 30 minutes (WT; n = 5 female, n = 6 male. CrT-OE; n = 11 female, n = 11 male). Hearts that did not meet minimum baseline function criteria were excluded from the study (1 male WT, 5 male CrT-OE and 4 female CrT-OE). In initial experiments, hearts were subjected to 20 minutes of global ischaemia, however hearts did not recover sufficiently, and therefore the ischaemic time was reduced. The aged heart has been previously shown to have an increased susceptibility to ischaemic time [16,17]. Unfortunately, LV creatine values were not available for all hearts in this study due to a problem with sample storage, therefore no correlation analysis was performed (hearts lost from WT; n = 7 and CrT-OE; n = 5).

### Cardioplegia

Hearts excised from WT and CrT-OE (30.6 ± 6 weeks) were stabilised for 15 minutes and arrested using St Thomas’ 2 cardioplegic solution (mM): NaCl (120), NaHCO_3_ (10), KCl (16), MgCl_2_.6H_2_O (16), CaCl_2_ (1.2) for 6 min (WT; n = 9 female, n = 7 male. CrT-OE; n = 8 female, n = 9 male). Hearts that did not meet the minimum function criteria during stabilisation were excluded from the study (2 male CrT-OE and 3 female CrT-OE). Hearts with the cannula intact were removed from the perfusion rig and submerged into cardioplegic solution for 90 minutes global ischaemia at 4°C. The intraventricular balloon was repositioned into the LV cavity and LVEDP reset to 5–10mmHg, hearts were reperfused for 30 minutes at 37°C.

### Statistical Analysis

All experiments and analysis were performed by a single perfusionist blind to the genotype and creatine levels. Data are expressed as mean ± SEM. Statistical comparison between two groups at a single time point was by Student’s t-test. A two-way repeated measures (mixed model) ANOVA with a Bonferroni correction for multiple comparisons was used to statistically compare groups at multiple time points. Differences were considered significant when p<0.05. All figures show data expressed as percentage of baseline (average of 3 stabilisation time points, i.e. first 15 min) with mean values for the raw data provided in the supporting information S1 File.

## Results

### Cardioprotection in presence of left ventricular hypertrophy

**Fig 1:**
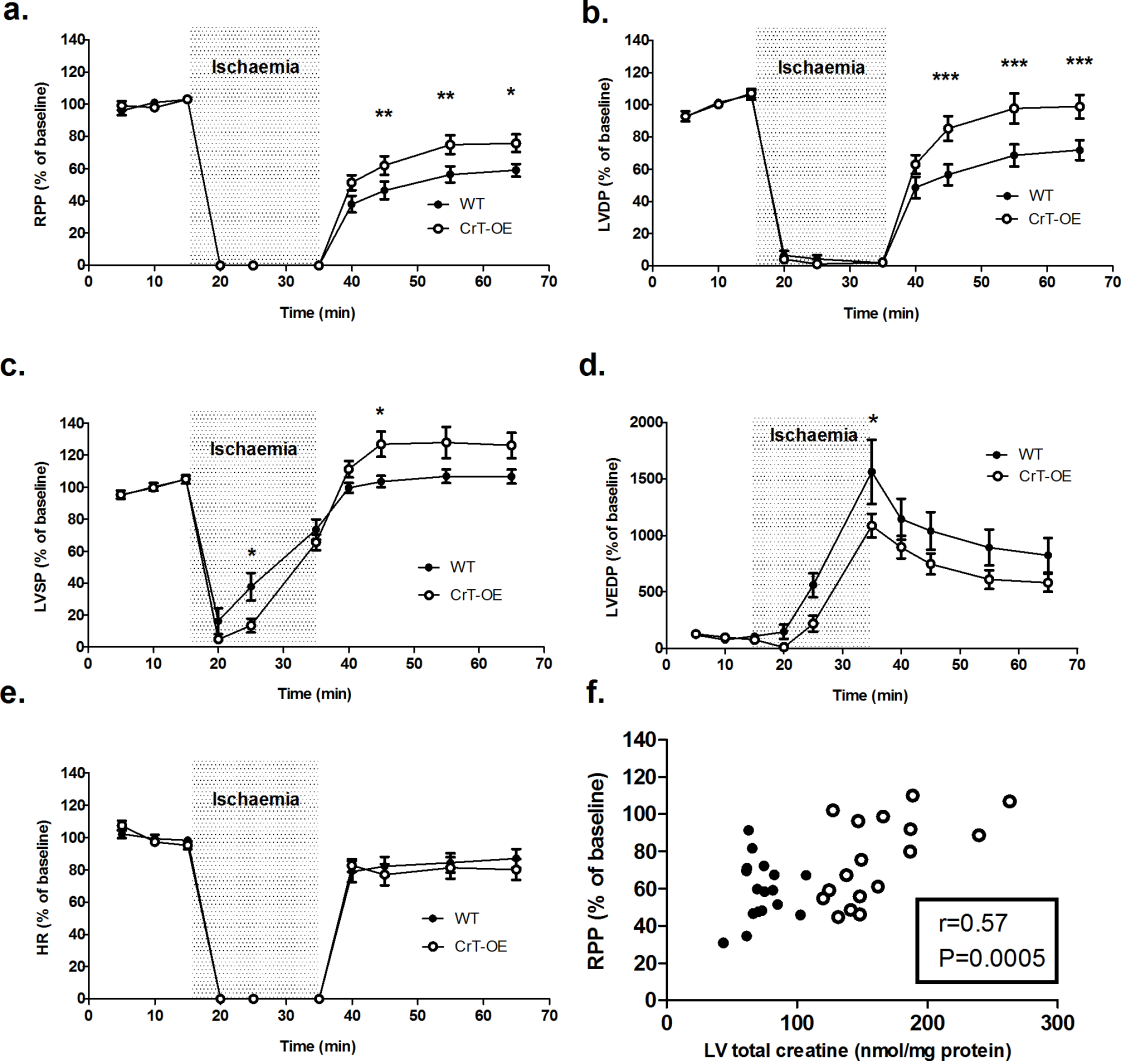
Functional recovery following ischaemia in hypertrophied hearts. Isolated hearts were perfused for 15 min at baseline, 20 min no flow ischaemia (grey) and 30 min reperfusion, with hypertrophied CrT-OE hearts (n = 17) showing improved functional recovery compared to hypertrophied WT hearts (n = 17). (a) rate pressure product, RPP; (b) left ventricular developed pressure, LVDP; (c) left ventricular systolic pressure, LVSP; (d) left ventricular end-diastolic pressure, LVEDP; (e) heart rate, HR. (f) Pearson's correlation analysis indicates a positive relationship between functional recovery and total creatine levels (WT hearts are black circles and CrT-OE white). Data shown as mean values ± SEM Comparisons between groups by two-way repeated measures (mixed model) ANOVA with a Bonferroni Post-hoc test. *P<0.05, **P<0.01, ***P<0.001.

A subset of WT mice (n = 4) received an echocardiogram prior to surgery and again at 10 days after TAC surgery. Compensated LVH was confirmed by a 27% increase in myocardial cross-sectional area (P = 0.02) in the absence of changes in FAC (P = 0.78). All other WT and CrT-OE mice were imaged at a single time-point approximately 10 days following TAC. There were no significant differences in echocardiographic measures of LVH or in vivo function (Table 1). Langendorff-perfused WT and CrT-OE hearts were subjected to 20 minutes global ischaemia and 30 minutes reperfusion. At baseline there were no significant differences in any functional parameters (Table A in S1 File)). During the last 20 minutes of reperfusion CrT-OE hearts had enhanced recovery of RPP and LVDP compared to WT hearts (Fig 1A and 1B). During ischaemia, no pressure is developed, so LVSP simply reflects diastolic pressure, which was higher in WT hearts suggesting greater ischaemic contracture (Fig 1D). Upon reperfusion, the recovery of LVSP was significantly higher (Fig 1C). These trends persisted throughout reperfusion, although did not reach statistical significance at later time-points. HR was unchanged in both groups (Fig 1E). LV creatine values correlated with recovery of cardiac function following ischaemia-reperfusion, Pearson r = 0.57, P = 0.0005 (Fig 1F).

**Table 1:** Cardiac function and physiological parameters following transverse aortic constriction.

	WT (17)		CrT-OE (17)	
Physiological parameters	*Male (8)*	*Female (9)*	*Male (8)*	*Female (9)*
*Body weight (g)*	*29*.*1 ± 1*.*0*	*23*.*7 ± 0*.*9*	*28*.*8 ± 0*.*9*	*23*.*5 ± 0*.*9*
*LV weight (mg)*	*151 ± 10*	*121 ± 7*	*164 ± 3*	*138 ± 6*
*LV/ BW ratio*	*5*.*19 ± 0*.*28*	*5*.*12 ± 0*.*29*	*5*.*72 ± 0*.*14*	*5*.*89 ± 0*.*22*
*LV total creatine (nmol/mg protein)*	*80 ± 6 (59–102)*	*66 ± 4 (43–82)*	185 ± 16*** (137–263)	142 ± 7*** (120–187)
**Cardiac Function (Echocardiography)**				
*HR (bpm)*	*488 ± 26*	*464 ± 16*	*494 ± 30*	*487 ± 15*
*Myocardial cross sectional area(mm*^*2*^*)*	*15*.*93 ± 0*.*83*	*12*.*32 ± 0*.*62*	*15*.*43 ± 0*.*54*	*13*.*16 ± 0*.*43*
*Fractional area change (%)*	*42*.*9 ± 6*.*0*	*38*.*1 ± 3*.*0*	*32*.*4 ± 3*.*3*	*33*.*5 ± 2*.*2*

Mean values ± SEM 10 days post TAC surgery for Echocardiography and 14 day post TAC for physiological parameters in WT and CrT-OE mice. ***P<0.001 versus WT mice

### Cardioprotection in old age

**Fig 2:**
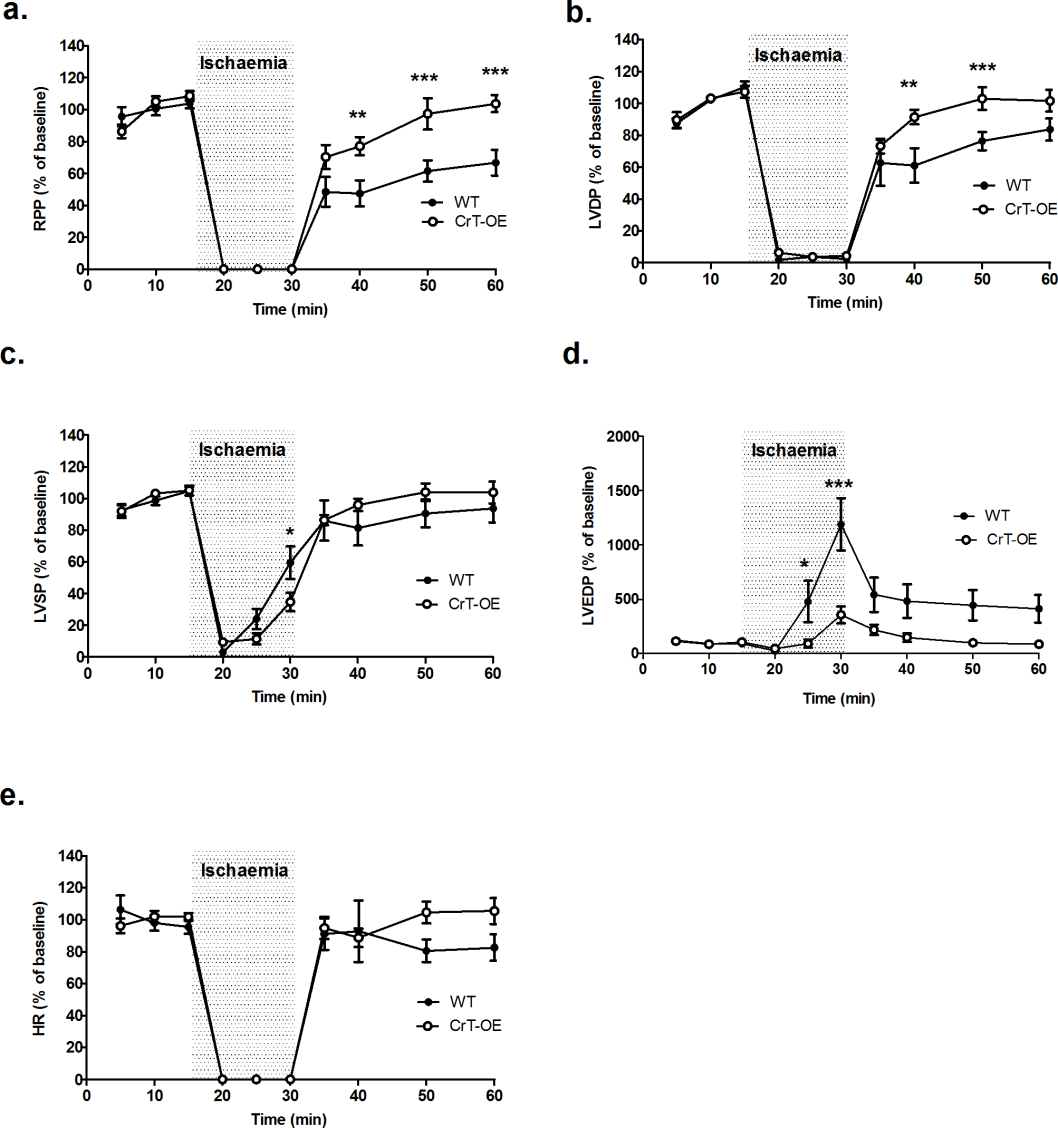
Functional recovery following ischaemia in ageing hearts. Isolated hearts were perfused for 15 min at baseline, 15 min no flow ischaemia (grey) and 30 min reperfusion, when hearts from aged CrT-OE mice (n = 13) had improved functional recovery compared to aged WT hearts (n = 10). (a) rate pressure product, RPP; (b) left ventricular developed pressure, LVDP; (c) left ventricular systolic pressure, LVSP; (d) left ventricular end-diastolic pressure, LVEDP; (e) heart rate, HR. Data shown as mean values ± SEM. Comparisons between groups by two-way repeated measures (mixed model) ANOVA with a Bonferroni Post-hoc test. *P<0.05, **P<0.01, ***P<0.001

Langendorff perfused hearts from aged CrT-OE mice (78 ± 5 weeks) had a significantly improved recovery of cardiac function (RPP) and developed pressure (LVDP) following 15 min ischaemia compared to aged WT hearts (Fig 2A & 2B). LV end-diastolic pressure increased to a greater extent during ischaemia in WT hearts but recovered to similar levels during reperfusion (Fig 2C & 2D). HR was unchanged in both groups (Fig 2E). There were no differences in baseline function between the groups (Table B in S1 File).

### Creatine elevation is additive to cardioplegic protection

**Fig 3:**
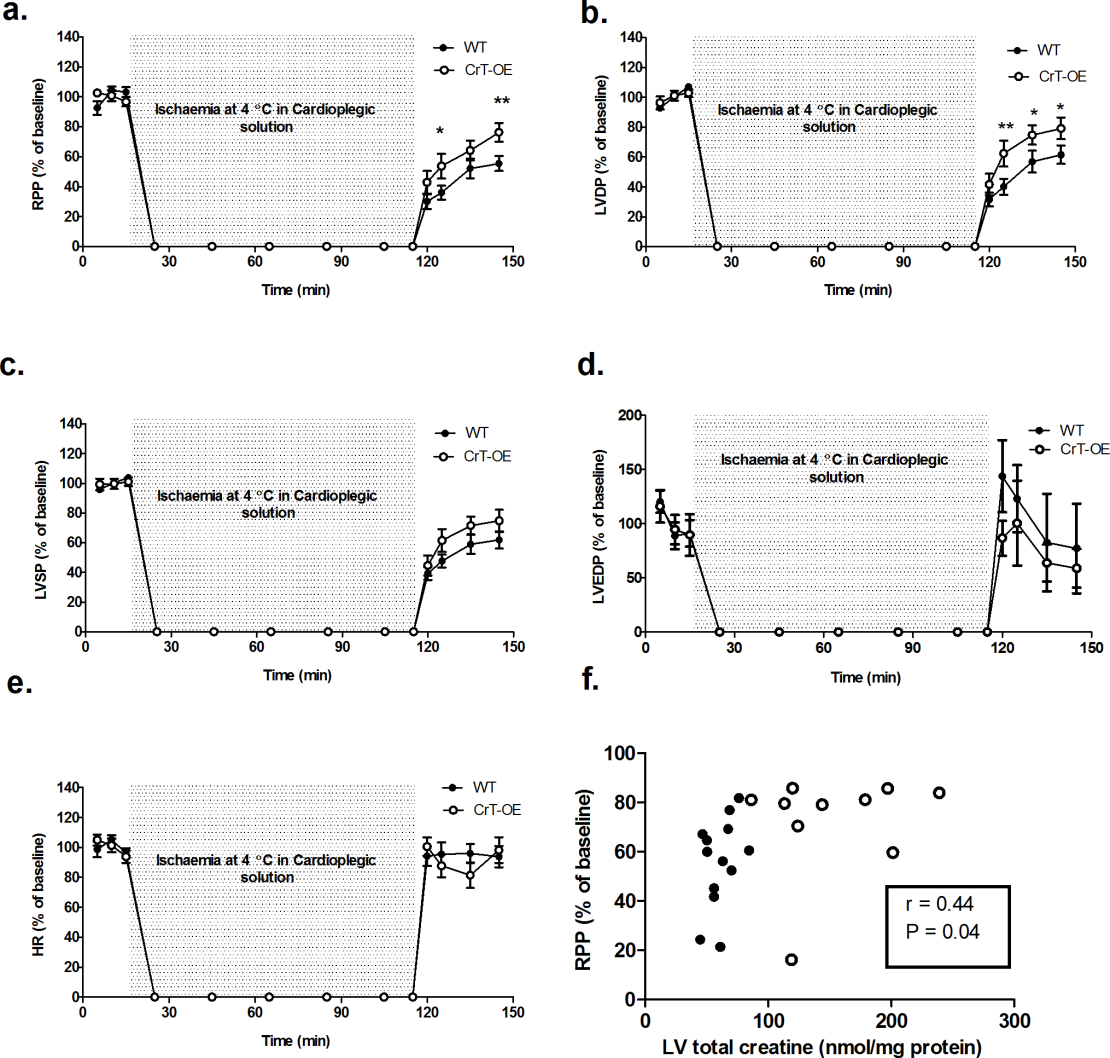
Functional recovery following hypothermic cardioplegic arrest. Isolated hearts were perfused for 15 min at baseline, then cardioplegic arrest for 90 min at 4°C (grey), and 30 min reperfusion, when CrT-OE hearts (n = 12) had improved functional recovery compared to WT hearts (n = 13). (a) rate pressure product, RPP; (b) left ventricular developed pressure, LVDP; (c) left ventricular systolic pressure, LVSP; (d) left ventricular end-diastolic pressure, LVEDP; (e) heart rate, HR. (f) Pearson's correlation analysis indicates a positive relationship between functional recovery and total creatine levels (WT hearts are black circles and CrT-OE white). Data shown as mean values ± SEM Comparisons between groups by two-way repeated measures (mixed model) ANOVA with a Bonferroni Post-hoc test. *P<0.05, **P<0.01

Cardioplegia-induced cardioprotection was enhanced in CrT-OE hearts subjected to 90 min ischaemia followed by reperfusion. RPP and LVDP were significantly improved during reperfusion, whereas all other functional parameters were not affected (Fig 3, raw data Table C in S1 File.) Of note, we took a conservative approach to exclude 1 male WT heart and 2 female WT hearts that did not recover following 90 min ischaemia from the analysis. Inclusion of these 3 data points strengthened the significance of the results. All CrT-OE hearts recovered and were included. Correlation analysis showed a relationship between LV total creatine and recovery of cardiac function, Pearson r = 0.44, P = 0.04.

## Discussion

This study builds upon previous work published by our laboratory that showed elevation of intracellular [Cr] by overexpression of the CrT protected young, healthy, mice against IRI, both in terms of cardiomyocyte survival in vivo and improved functional recovery ex vivo. This was attributed to increased phosphocreatine (PCr) buffer capacity, elevated myocardial glycogen, improved ‘energy reserve’ and the ability of creatine to reduce the open probability of the mitochondrial permeability transition pore [6]. Cardioprotective strategies that are successful in the laboratory frequently get ‘lost in translation’ when trialled in the clinic [18], and in the current study, we therefore sought to extend these observations into mice with pre-existing comorbidities. We demonstrate for the first time that elevating intracellular [Cr] retains its beneficial effect in aged and compensated hypertrophied hearts. Moreover increasing myocardial [Cr] enhances the protective effect already afforded by cardioplegia during prolonged ischaemia.

Rapid revascularization of ischaemic regions within the heart by thrombolysis or primary percutaneous coronary intervention remains at the clinical forefront to effectively treat AMI [18,19]. Pre-existing hypertrophy has been associated with a significantly worse prognosis in patients recovering from AMI [20,21]. The deleterious effects of ischaemia may be more pronounced in the hypertrophied heart, in part because the capillary network is unaltered despite increased myocyte volume (and metabolic requirements), resulting in a greater distance for oxygen diffusion [22]. This phenomenon has also been observed in the murine TAC model [23], which we used at two weeks post-surgery since we have previously shown this to result in robust hypertrophy without deterioration in cardiac function [14]. Major differences in starting baseline function would have made our results difficult to interpret. We observed greater functional recovery following ischaemia in CrT-OE hearts despite slightly more pronounced LVH and a trend for lower baseline in vivo function.

It should be noted that previous work from our laboratory has shown that very high myocardial [Cr] may itself cause LV hypertrophy and dysfunction [12], and defined the safe “therapeutic” level at up to 100% above wild-type [6]. Since we did not prospectively exclude very high [Cr] levels in the current experiments, the higher proportion of transgenic mice excluded in the ageing and cardioplegia studies may simply reflect those animals with pathologically high levels of [Cr] (i.e. >100% above WT). It is conceivable that very high [Cr] may be detrimental in the long-term, but remain beneficial in the context of acute ischaemia. This is supported by the positive linear correlations we observed between [Cr] and functional recovery, particularly in the TAC experiment which included a number of hearts within the high (detrimental) [Cr] range. Since the adverse effects of high [Cr] are driven by an inability to keep the total creatine pool adequately phosphorylated [12], we speculate that it may be possible to extend the “therapeutic” range by simultaneously increasing activity of creatine kinase (CK). In this context it is notable that over-expression of CK in mouse heart also protects against IRI [24].

We also performed experiments in mice aged 18 months since the ageing heart is known to have altered cardiac substrate metabolism [25], is more susceptible to ischaemic damage [26], and is less amenable to conditioning strategies [27,28]. The maturational rate in mice ranges from 150x faster than humans in the first month of life to 25x faster beyond 6 months. By 18 months, virtually all biomarkers of ageing are evident and mice are classed as old age, equivalent to 56–69 years in humans [29]. This corresponds well with the median age for first AMI, which is 56 years for men and 65 years for women [30].

Cold, cardioplegic arrest of the heart remains the gold standard for effective myocardial protection during cardiac surgery [31] and heart transplantation [32], however the heart is not completely protected against ischaemic damage. Cold St Thomas’ solution #2 protects the myocardium via hypothermia and electromechanical arrest using high concentrations of K^+^ and Mg^2+^ ions to reduce metabolic demand and prolong ischaemic tolerance [31]. In this context, elevation of intracellular [Cr] had an additive cardioprotective effect. We took the conservative approach of excluding three wild-type hearts that failed to show any recovery upon reperfusion, so this effect size may be an underestimation. Previous studies have shown that supplementation of cardioplegic solutions with exogenous phosphocreatine can significantly improve myocardial protection, e.g. in pigs [33] and in patients [34] undergoing coronary artery bypass surgery, and in *ex vivo* rat hearts [35]. However, it should be noted that phosphocreatine is not a substrate for cellular uptake via the CrT [36]. These beneficial effects are proposed to result from enhanced plasma membrane stabilisation [37] and not elevation of intracellular [Cr] as in the current study. The capability to enhance protection above that already reported for cardioplegia alone is an important finding that could translate into the clinical environment to improve patient outcomes following cardiac surgery.

### Limitations

During ischaemia, the heart undergoes many metabolic changes including a reduction in intracellular PCr [38]. We did not measure PCr in this study but from previous studies we know that myocardial PCr levels were on average 49% higher in CrT-OE mice [6]. Instead we measured total [Cr] which includes PCr using HPLC at the end of perfusion experiments. WT values were comparable to historical values for freshly harvested LV tissue confirming total [Cr] was not lost over the experimental timeframe. The findings presented in this study are from *ex vivo* not *in vivo* experimental approaches and ideally we would have both. This reflects the limitations of having sufficient numbers of aged animals and the welfare implications of combining TAC surgery with ischaemia-reperfusion surgery. Furthermore, we did not perform tetrazolium staining in our ex vivo hearts since the period of reperfusion was insufficient for complete washout of NADH from necrotic tissue, which would lead to under-estimation of infarct sizes [39]. It is therefore not possible for us to determine whether the protective effect of elevating creatine was due to increased cell survival or improved contractile function. However, our previous study showed that both aspects play a role in cardioprotection in CrT-OE mice [6].

## Conclusions

We have shown that elevating creatine levels in the mouse heart improves functional recovery following ischaemia-reperfusion even in the presence of old age or LVH. Both conditions previously associated with loss of cardioprotective efficacy. Furthermore, elevating intracellular creatine is additive to standard hypothermic cardioplegia for recovery from prolonged ischaemia. Together these findings support the development of small molecule activators that increase intracellular Cr [40], which would be necessary for testing in large animal models and ultimately translation to the clinic. Such compounds would increase myocardial [Cr] with potential for beneficial effects when given prior to elective cardioplegic surgery, donor explant or in patients deemed at high risk of AMI.

## Supporting Information

S1 File Tables A-C. (DOC)
