# Supplementary material for: Protective Effect of Creatine Elevation against Ischaemia Reperfusion Injury Is Retained in the Presence of Co-Morbidities and during Cardioplegia
Source: PLoS One. 2016 Jan 14;11(1):e0146429. doi: 10.1371/journal.pone.0146429 (PMC4713158; doi:10.1371/journal.pone.0146429)
Supplement: S1 File — (DOC) [file pone.0146429.s001.doc]

|  | **WT (17)**  *(male 8, female 9)* | | | | | **CrT-OE (17)**  *(male 8, female 9)* | | | | |
| --- | --- | --- | --- | --- | --- | --- | --- | --- | --- | --- |
|  | **Functional Parameters (*Ex vivo*)** | | | | | | | | | |
| ***Minutes*** | **RPP**  *mmHg * bpm* | **LVDP**  *mmHg* | **LVSP** *mmHg* | **LVEDP** *mmHg* | **HR** *bpm* | **RPP**  *mmHg *bpm* | **LVDP**  *mmHg* | **LVSP**  *mmHg* | **LVEDP** *mmHg* | **HR**  *bpm* |
| ***Baseline*** |  |  |  |  |  |  |  |  |  |  |
| *15* | 25981 ± 1355 | 88.8 ± 6.9 | 94.1 ± 6.9 | 5.6 ± 0.8 | 312 ± 19 | 25079± 1278 | 84.0 ± 4.5 | 89.4 ± 4.7 | 5.7 ± 0.8 | 309 ± 18 |
| ***Global Ischaemia*** |  |  |  |  |  |  |  |  |  |  |
| *20* | 0 | 1.6 ± 0.5 | 65.4 ± 5.5 | 63.8 ± 5.3 | 0 | 0 | 1.8 ± 0.4 | 56.6 ± 3.6 | 54.9 ± 3.7 | 0 |
| ***Reperfusion*** |  |  |  |  |  |  |  |  |  |  |
| *5* | 10247 ± 1623 | 43.3 ± 6.4 | 92.4 ± 5.6 | 50.5 ± 4.4 | 244 ± 23 | 12656 ± 1122 | 54.1 ± 6.3 | 98.5 ± 6.0 | 44.3 ± 3.3 | 251 ± 15 |
| *10* | 12486 ± 1772 | 50.6 ± 6.5 | 96.2 ± 6.2 | 46.0 ± 4.5 | 257 ± 24 | 15325 ± 1449 | 72.3 ± 7.6 | 112.3 ± 8.4 | 37.1 ± 3.2 | 234 ± 15 |
| *20* | 14917 ± 1672 | 59.9 ± 6.5 | 98.8 ± 6.1 | 38.6 ± 4.1 | 259 ± 21 | 18164 ± 1101 | 80.9 ± 7.0 | 111.3 ± 7.2 | 30.8 ± 3.2 | 241 ± 17 |
| *30* | 15366 ± 1371 | 62.3 ± 6.0 | 98.3 ± 6.0 | 35.4 ± 3.9 | 264 ± 18 | 18336 ± 1030 | 82.8 ± 7.1 | 110.9 ± 7.6 | 29.0 ± 2.9 | 238 ± 17 |

Table A. Left ventricular hypertrophy. Ex vivo functional raw data in WT and CrT-OE mice following transverse aortic constriction for 2 weeks, subjected to 20 min ischaemia and 30 min reperfusion. Data shown as mean values ± SEM.

|  | **WT (10)**  *(male 5, female 5)* | | | | | **CrT-OE (13)**  *(male 6, female 7)* | | | | |
| --- | --- | --- | --- | --- | --- | --- | --- | --- | --- | --- |
|  | **Functional Parameters (Ex vivo)** | | | | | | | | | |
| ***Minutes*** | **RPP**  *mmHg* bpm* | **LVDP** *mmHg* | **LVSP** *mmHg* | **LVEDP** *mmHg* | **HR** *bpm* | **RPP**  *mmHg**  *bpm* | **LVDP** *mmHg* | **LVSP** *mmHg* | **LVEDP** *mmHg* | **HR**  *bpm* |
| ***Baseline*** |  |  |  |  |  |  |  |  |  |  |
| *15* | 19120 ± 1680 | 60.2 ± 5.7 | 67.1 ± 6.2 | 4.8 ± 0.7 | 324 ± 24 | 17244 ± 1440 | 56.8 ± 5.4 | 60.1 ± 5.4 | 4.9 ± 0.8 | 290 ± 18 |
| ***Global Ischaemia*** |  |  |  |  |  |  |  |  |  |  |
| *15* | 0 | 1.4 ± 0.2 | 39.5 ± 6.4 | 44.0 ± 5.6 | 0 | 0 | 2.9 ± 1.0 | 22.3 ± 4.5 | 18.3 ± 4.1 | 0 |
| ***Reperfusion*** |  |  |  |  |  |  |  |  |  |  |
| *5* | 8663 ± 1540 | 34.2 ± 7.4 | 54.0 ± 6.4 | 20.2 ± 3.8 | 290 ± 32 | 11187 ± 1493 | 40.6 ± 3.5 | 51.7 ± 3.8 | 11.3 ± 2.3 | 276 ± 23 |
| *10* | 8962 ± 1508 | 35.3 ± 6.0 | 52.5 ± 6.3 | 17.4 ± 3.4 | 279 ± 41 | 12142 ± 1062 | 51.0 ± 4.5 | 58.1 ± 5.2 | 7.7 ± 1.9 | 258 ± 19 |
| *20* | 11496 ± 1337 | 44.5 ± 3.2 | 58.8 ± 4.9 | 15.6 ± 3.1 | 256 ± 23 | 15430 ± 1882 | 56.6 ± 4.8 | 62.0 ± 4.9 | 4.9 ± 1.4 | 294 ± 12 |
| *30* | 12642 ± 1698 | 48.8 ± 4.1 | 60.8 ± 5.3 | 14.3 ± 2.8 | 263 ± 29 | 16703 ± 1585 | 55.5 ± 4.3 | 61.8 ± 5.2 | 4.2 ± 1.4 | 294 ± 10 |

Table B. Old Age. Ex vivo functional raw data in aged WT and CrT-OE mice (78 ± 5 weeks) subjected to 15 min ischaemia and 30 min reperfusion. Data shown as mean values ± SEM.

|  | **WT (13)**  *(male 6, female 7)* | | | | | **CrT-OE (12)**  *(male 7, female 5)* | | | | |
| --- | --- | --- | --- | --- | --- | --- | --- | --- | --- | --- |
|  | **Functional Parameters (*Ex vivo*)** | | | | | | | | | |
| *Minutes* | **RPP**  *mmHg*bpm* | **LVDP**  *mmHg* | **LVSP** *mmHg* | **LVEDP** *mmHg* | **HR** *bpm* | **RPP** *mmHg* bpm* | **LVDP** *mmHg* | **LVSP** *mmHg* | **LVEDP**  *mmHg* | **HR**  *bpm* |
| ***Baseline*** |  |  |  |  |  |  |  |  |  |  |
| *15* | 22590 ± 1557 | 65.9 ± 3.0 | 71.5 ± 3.2 | 6.1 ± 0.9 | 350 ± 23 | 22482 ± 1071 | 75.0 ± 5.1 | 76.7 ± 5.1 | 5.3 ± 0.8 | 317 ± 20 |
| **Global Ischaemia for 90 minutes in cardioplegic solution at 4°C** | | | | | | | | | | |
| ***Reperfusion*** |  |  |  |  |  |  |  |  |  |  |
| *5* | 6500 ± 994 | 19.8 ± 2.8 | 28.5 ± 3.6 | 7.4 ± 1.3 | 325 ± 25 | 9565 ± 1561 | 31.0 ± 5.0 | 33.2 ± 4.7 | 4.1 ± 0.7 | 316 ± 20 |
| *10* | 7902 ± 893 | 26.0 ± 3.3 | 34.3 ± 3.6 | 7.2 ± 1.5 | 323 ± 22 | 11891 ± 1767 | 45.9 ± 6.7 | 46.8 ± 6.4 | 4.4 ± 1.3 | 278 ± 25 |
| *20* | 11531 ± 1361 | 36.5 ± 4.3 | 42.9 ± 5.1 | 3.5 ± 1.3 | 328 ± 21 | 14100 ± 1767 | 55.6 ± 5.1 | 53.7 ± 4.9 | 3.6 ± 1.1 | 263 ± 30 |
| *30* | 12306 ± 1132 | 40.6 ± 3.9 | 44.5 ± 4.4 | 2.9 ± 1.1 | 324 ± 27 | 16762 ± 1278 | 56.6 ± 4.6 | 55.0 ± 4.7 | 3.3 ± 1.1 | 305 ± 23 |

Table 3. Cardioplegia. *Ex vivo* functional raw data in WT and CrT-OE mice. Hearts were arrested for 6 min with perfusion of 'St Thomas' cardioplegic solution 2' and kept submerged in solution for a duration of 90 minutes global ischaemia at 4°C. Hearts were reperfused for 30 minutes with Krebs-Henseleit buffer and recovery monitored. Data shown as mean values ± SEM.
